# Interobserver reliability is higher for assessments with 3D software-generated models than with conventional MRI images in the classification of trochlear dysplasia

**DOI:** 10.1007/s00167-021-06697-3

**Published:** 2021-08-22

**Authors:** Andreas Fuchs, Matthias J. Feucht, Jörg Dickschas, Jannik Frings, Markus Siegel, Tayfun Yilmaz, Hagen Schmal, Kaywan Izadpanah

**Affiliations:** 1 Department of Orthopedic Surgery and Traumatology, Freiburg University Hospital, Albert Ludwigs University Freiburg, Hugstetter Straße 55, 79106 Freiburg, Germany; 2 Orthopädische Klinik Paulinenhilfe, Diakonieklinikum Stuttgart, Rosenbergstr. 38, 70176 Stuttgart, Germany; 3 Klinik Für Orthopädie Und Unfallchirurgie, Klinikum Bamberg, Buger Strasse 80, 96049 Bamberg, Germany; 4 Department of Trauma and Orthopaedic Surgery, University Medical Center Hamburg-Eppendorf, Martinistraße 52, 20246 Hamburg, Germany

**Keywords:** Trochlear dysplasia, Patellar instability, Dejour, 3D model

## Abstract

**Purpose:**

Trochlear dysplasia is a significant risk factor for patellofemoral instability. The severity of trochlear dysplasia is commonly evaluated based on the Dejour classification in axial MRI slices. However, this often leads to heterogeneous assessments. A software to generate MRI-based 3D models of the knee was developed to ensure more standardized visualization of knee structures. The purpose of this study was to assess the intra- and interobserver agreements of 2D axial MRI slices and an MRI-based 3D software generated model in classification of trochlear dysplasia as described by Dejour.

**Methods:**

Four investigators independently assessed 38 axial MRI scans for trochlear dysplasia. Analysis was made according to Dejour’s 4 grade classification as well as differentiating between 2 grades: low-grade (types A + B) and high-grade trochlear dysplasia (types C + D). Assessments were repeated following a one-week interval. The inter- and intraobserver agreement was determined using Cohen’s kappa (κ) and Fleiss kappa statistic (κ). In addition, the proportion of observed agreement (po) was calculated for assessment of intraobserver agreement.

**Results:**

The assessment of the intraobserver reliability with regard to the Dejour-classification showed moderate agreement values both in the 2D (κ = 0.59 ± 0.08 SD) and in the 3D analysis (κ = 0.57 ± 0.08 SD). Considering the 2-grade classification, the 2D (κ = 0.62 ± 0.12 SD) and 3D analysis (κ = 0.61 ± 0.19 SD) each showed good intraobserver matches. The analysis of the interobserver reliability also showed moderate agreement values with differences in the subgroups (2D vs. 3D). The 2D evaluation showed correspondences of κ = 0.48 (Dejour) and κ = 0.46 (high / low). In the assessment based on the 3D models, correspondence values of κ = 0.53 (Dejour) and κ = 0.59 (high / low) were documented.

**Conclusion:**

Overall, moderate-to-good agreement values were found in all groups. The analysis of the intraobserver reliability showed no relevant differences between 2 and 3D representation, but better agreement values were found in the 2-degree classification. In the analysis of interobserver reliability, better agreement values were found in the 3D compared to the 2D representation. The clinical relevance of this study lies in the superiority of the 3D representation in the assessment of trochlear dysplasia, which is relevant for future analytical procedures as well as surgical planning.

**Level of evidence:**

Level II.

## Introduction

Trochlear morphology is recognized as one of the most important factors for patellar stability [6, 9, 11, 18, 19, 26]. Trochlear dysplasia is estimated to occur in less than 2% of the general population, whereas, 62%–96% of patients with patellar instability have evidence of trochlear dysplasia [5, 6, 10]. The severity of trochlear dysplasia is commonly evaluated and categorized based on the Dejour radiographic and magnetic resonance imaging (MRI) classifications [9, 12, 15]. In the past decades MRI has become the standard to assess patellofemoral instability [1, 4, 8, 20, 22, 25]. On axial MRI, trochlear dysplasia is diagnosed on the first craniocaudal image, where the complete cartilaginous trochlea can be seen. Dejour classified trochlear dysplasia into type A (fairly shallow trochlea), type B (flat or convex trochlea), type C (asymmetry of trochlear facets with a hypoplastic medial condyle), and type D (asymmetry of trochlear facets plus vertical join and cliff pattern) [5]. However, poor inter- and intraobserver agreement values using Dejour’s 4 type classification has been reported in evaluation of axial MRI [12, 13, 15, 27] with low correlation to objective intraoperative findings [14].

Due to the complex surface of anatomy observed in trochlear dysplasia, the best possible and standardized visualization of the bony and cartilage structures is of crucial importance. Here, 3D imaging possibly offers good options. It allows to present the individual anatomy in a physical three-dimensional model and potentially eases the capture of spatial proportions, especially in cases of complex anatomy [7]. For this reason, an MRI-based 3D model of the knee was created, in order to enable an improved analysis of the complex anatomical conditions and thus a more reliable therapy planning in the future. The aim of this study was to assess the intra- and interobserver agreements of 2D axial magnetic resonance images and an MRI-based 3D software-generated model in classification of trochlear dysplasia and to compare the commonly used Dejour’s 4-grade-classication system with a 2-grade-classification system based on Dejour’s classification. It was hypothesized that there are better agreement values in 3D compared to 2D representation.

## Materials and methods

A retrospective evaluation of 38 MRI scans of 38 patients with trochlear dysplasia was performed. This retrospective study was approved by an institutional review board (Technical University Munich, ID-number: 208/10 S-KK). The selection of the MRI scans was made at random by one of the authors out of 80 patients who had undergone patellofemoral stabilization surgery within a year without reference to the extent of the trochlear dysplasia present. Patients with previous surgery on bony structures of the knee or MRI images of poor quality (< 1.5 Tesla) were excluded. The allocation of the 38 knee joints with trochlear dysplasia according to Dejour (Consensus of all investigators after independent repeated classification of trochlea types—the maximum agreement of the independent classification of all investigators was decisive for the consensus) showed the following distribution: type A: *n* = 13, type B: *n* = 14, type C: *n* = 5, type D: *n* = 6. None of the patients had any history of knee surgery altering the form of the femoral trochlea prior to MRI.

Four orthopedic surgeons independently graded the trochlear shape in axial T2-weighted MRI slices of the most proximal transverse MRI where the cartilage along the entire width of the trochlea was visible. Each MRI was performed in normal clinical routine with the patient in a supine position. Due to the acquisition of patients in normal clinical routine, the MRI images were produced on different devices in different institutions. Inclusion criteria for use within this study were: MRI ≥ 1.5 Tesla, representation of the entire trochlea, no movement artifacts and no metal artifacts. Afterwards, all four surgeons repeated the assessment and classification on MRI based 3D image-models of the same patients. Both in the assessment of axial MRI slices and in the assessment of 3D models, it was possible to switch freely between the slices or views. The standard 3D view showed both bone and cartilage tissue, but it was also possible to temporarily hide the cartilage structures. Both assessments were repeated by all surgeons after a minimum interval of one week. The order of the cases was randomized to eliminate any memory bias.

The 3D models were generated by slice-wise segmentation of the 38 MRI scans. Segmentation of bone and cartilage was initially done manually for the whole cohort in a web-based application (Fraunhofer MEVIS Knee SATORI, Version 1.0.0a). Several positions inside and outside the respective structure were manually marked until the corresponding segmentation, which was updated in real time, highlighted the correct extent. The resulting surface was postprocessed by snapping it to a subvoxel precise position based on a cubic interpolation of the image data. The results of the manual segmentations of all subjects were used to train the web application in segmentation for the different structures. The trained model was then applied to the same images for more consistent segmentation results. After segmentation (manual or automatic), the 3D models are created immediately using the web application.

**Fig. 1:**
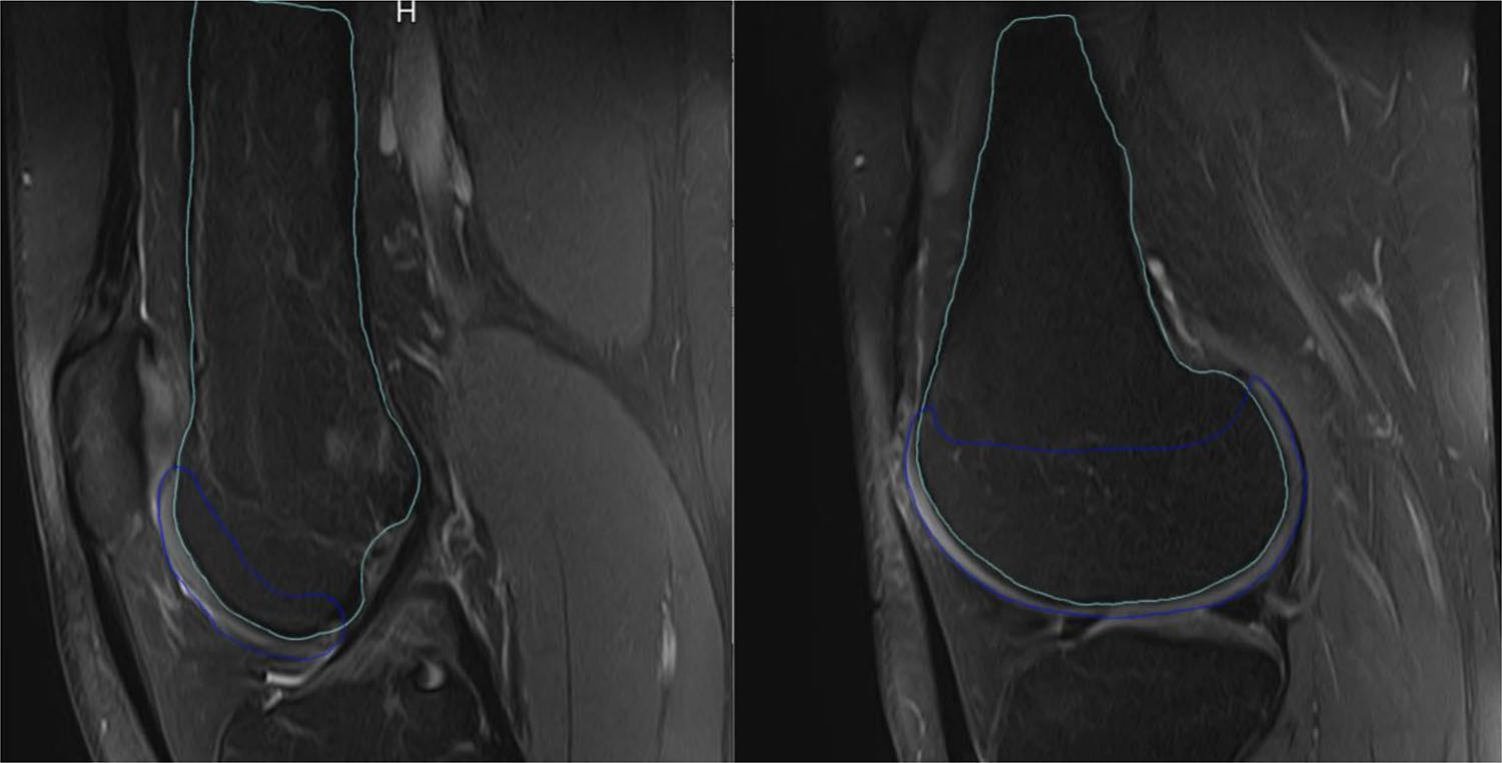
Segmentation of cartilage (blue) and bone (green) structures of the distal femur in T2 weighted sagittal MRI slices. *Blue line: segmentation of femoral cartilage in sagittal MRI slices for the creation of a 3D model; Green line: segmentation of femoral bone in sagittal MRI slices for the creation of a 3D model

**Fig. 2:**
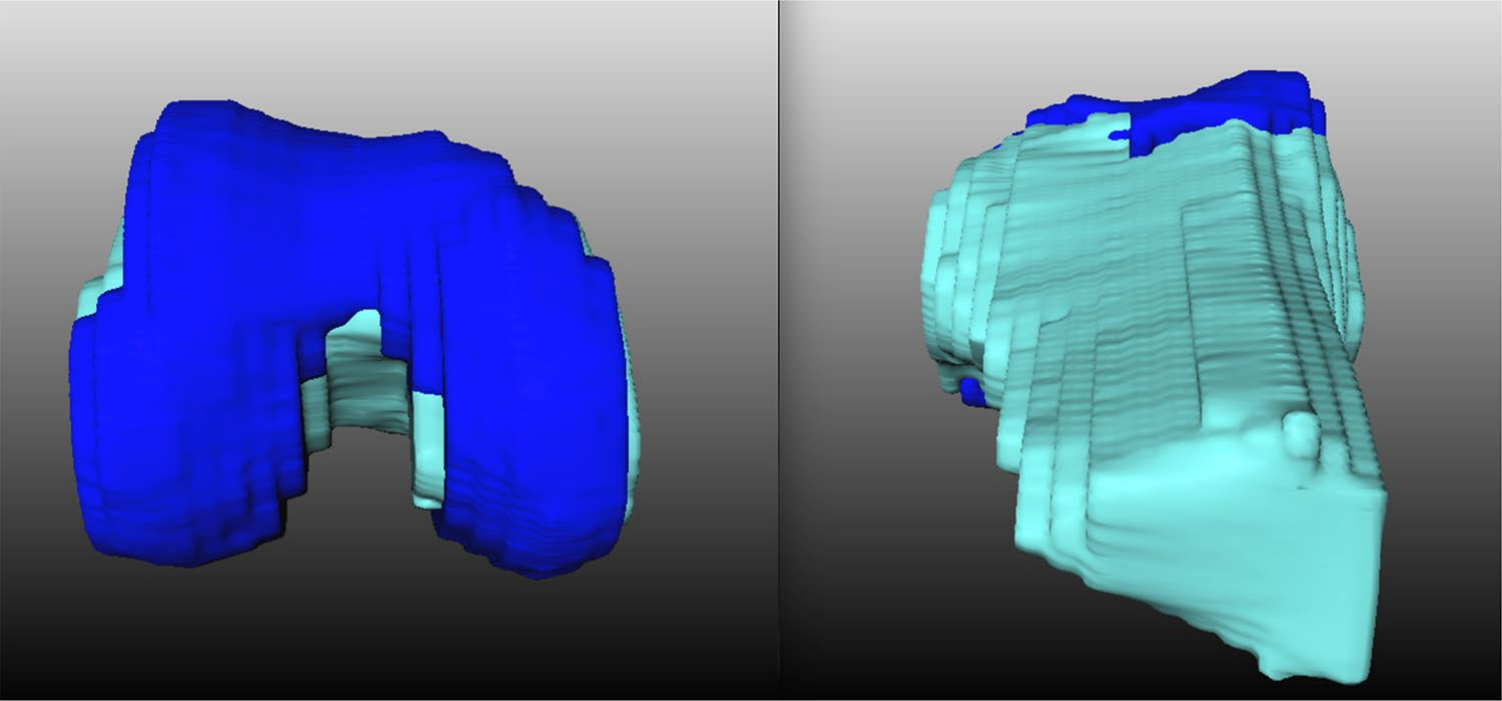
Software-generated MRI-based 3D model of the knee. **Blue structure: representation of the femoral cartilage in a 3D model; Green structure: representation of the femoral bone in a 3D model; *Left* camera orientation: foot, *Right* camera orientation: head

The results were first analyzed with regards to the four classification types (Dejour) between the four readers. For further investigation, a differentiation in a 2-grade classification system was additionally chosen. Here, the subgroups “Low grade” dysplasia (Dejour A + B) and “High grade” dysplasia (Dejour C + D) were investigated Figs 1 and 2.

### Statistical analysis

The intraobserver/intermethod agreement was determined using Cohen’s kappa statistic. Interobserver agreement was assessed using Fleiss kappa statistic. The kappa statistic expresses the chance-corrected agreement. It is the (normalized) observed agreement minus the agreement expected on the basis of chance alone. The expected agreement is based on the prevalence of each grade, which was calculated from the combined ratings of all raters. A kappa value greater than 0.75 represents excellent agreement, values between 0.60 and 0.74 good, values between 0.40 and 0.59 moderate, values between 0.21 and 0.40 slight and values below 0.20 poor agreement [24].

In addition, the proportion of observed agreement (po) was calculated, including 95% confidence interval (CI), for assessment of intraobserver agreement. The proportion p_0_ describes the percentage of agreement and is calculated from the sum of the observed absolute frequencies in the main diagonal of contingency tables divided by the total number of patients.

Three different investigations were performed separately for the 4-graded classification as well as the 2-graded classification:

1. the intraobserver agreement was investigated (agreement of ratings made at the first and second time points of assessment: for each rater, 2D and 3D, Cohens Kappa and proportion of agreement).
2. the intermethod agreement of 2D and 3D analysis was investigated (for each rater, for each time point of assessment, Cohens Kappa and proportion of agreement).
3. the interobserver agreement was investigated (a comparison of all raters for each time point of assessment, 2D and 3D, Fleiss Kappa).

Statistical analyses were carried out using IBM SPSS Statistics Version 27.0.0.0 (IBM Corp., Armonk, New York). The results of all statistical tests were interpreted in an exploratory sense.

## Results

In the following, relevant results are summarized in Kappa values (κ) and proportion of agreement (p_0_).

### Intraobserver agreement, 4-grade analysis

When observers classified trochlear dysplasia into Dejour’s four grades, the intraobserver agreement in 2D MRI evaluation between the first and the second reading was 65–78%. The mean Kappa value (Cohens Kappa) was 0.59 (SD ± 0.08). Intraobserver agreement for classification into four grades for the two readings of the 3D models was 63–74%. The mean Kappa value (Cohens Kappa) was 0.57 (SD ± 0.08) (Table 1).

**Table 1:** Intraobserver agreement, 2-grade- and 4-grade analysis*

Rater	4-grade analysis (Dejour)		2-grade analysis (High/Low)	
	2D	3D	2D	3D
*Cohen’s Kappa*				
1	0.59	0.66	0.58	0.82
2	0.71	0.49	0.77	0.71
3	0.57	0.54	0.78	0.51
4	0.51	0.60	0.36	0.41
*Proportion of agreement*				
1	69% (54;84)	74% (60;88	81% (68;94)	92% (83;100)
2	65% (50;80)	71% (56;86)	70% (55;85)	70% (55;85)
3	78% (65;91)	63% (47;79)	89% (79;99)	87% (76;98)
4	68% (53;83)	64% (49;79)	89% (79;99)	76% (62;90)

^*^4-grade analysis, Dejour classification; 2-grade analysis, high/low grade trochlear dysplasia; 2D, 2D axial MRI scan; 3D, MRI-based 3D model; values in parenthesis represent 95% confidence interval

### Intraobserver agreement, 2-grade-analysis

The intraobserver agreement in the evaluation of the subgroups low-grade dysplasia (Dejour A + B) and high-grade dysplasia (Dejour C + D) between the first and the second reading was 70–89% in 2D- and 70–92% in 3D-analysis. The mean Kappa values (Cohens Kappa) were 0.62 (SD ± 0.12) for 2D- and 0.61 (SD ± 0.19) for 3D- assessment. (Table 1).

### Intermethod agreement of 2D- and 3D-evaluation, 4-grade analysis

Using the 4-grade classification according to Dejour, the agreement of 2D and 3D evaluation at the first reading was 41–57%. At the second reading, an agreement between 41 and 59% was achieved. These findings correspond to mean Kappa-values (Cohens Kappa) of κ = 0.32 (SD ± 0.09) for the first reading and κ = 0.35 (SD ± 0.15) for the second reading (Table 2).

**Table 2:** Intermethod agreement, 2-grade- and 4-grade analysis**

Rater	4-grade analysis (Dejour)		2-grade analysis (High/Low)	
	t1	t2	t1	t2
*Cohen’s Kappa*				
1	0.41	0.40	0.76	0.51
2	0.33	0.45	0.48	0.66
3	0.34	0.42	0.29	0.35
4	0.19	0.12	0.27	0.30
*Proportion of agreement*				
1	57% (41;73)	57% (41;73)	89% (79;99)	78% (65;91)
2	41% (50;80)	41% (25;57)	65% (50;80)	65% (50;80)
3	51% (65;91)	59% (43;75)	76% (62;90)	84% (72;96)
4	51% (53;83)	57% (41;73)	65% (50;80)	68% (53;83)

^**^4-grade analysis, Dejour classification; 2-grade analysis, high/low grade trochlear dysplasia; t1, time point 1; t2, time point 2; values in parenthesis represent 95% confidence interval

### Intermethod agreement of 2D- and 3D-evaluation, 2-grade analysis

For the 2-grade classification, the agreement of 2D and 3D evaluation at the first reading ranged from 65 to 89%. At the second reading, an agreement of 65–84% was ascertained. The evaluation of agreement according to Cohens-Kappa statistics showed mean κ = 0.45 (SD ± 0.23) for the first and κ = 0.45 (SD ± 0.16) for the second reading (Table 2).

### Interobserver agreement, 4-grade analysis

The overall Interobserver agreement in the analysis of the 4-grade classification was κ = 0.48 for 2D and κ = 0.53 for 3D evaluation (Table 3).

**Table 3:** Interobserver agreement, 2-grade- and 4-grade analysis***

	2D			3D		
	All	t1	t2	All	t1	t2
4-grade (Dejour)	0.48	0.44	0.48	0.53	0.58	0.47
2-grade (High/Low)	0.46	0.43	0.40	0.59	0.63	0.57

^***^Fleiss-Kappa values of Interrater agreement; 4-grade, Dejour classification; 2-grade, high/low grade trochlear dysplasia; 2D, 2D axial MRI scan; 3D, MRI-based 3D model; t1, time point 1; t2, time point 2

### Interobserver agreement, 2-grade analysis

The overall Interobserver agreement in the analysis of the 2-grade classification was κ = 0.46 for 2D and κ = 0.59 for 3D evaluation (Table 3).

## Discussion

The most important finding of this study is that data of interobserver reliability show better agreement values in the assessment of the 3D models compared to conventional MRI images, both, in the 4-degree as well as in the 2-degree classification. Although an absolute comparison of the Fleiss kappa values in the statistical analysis is not tenable due to different statistical assumptions, the comparison of the agreement values shows a slight superiority of the 3D representation in the interobserver agreement. Further findings were that the analysis of intraobserver reliability showed no relevant differences between 2 and 3D representation, but better agreement values were found in the 2-degree classification compared to the 4-degree classification.

Trochlear morphology is a highly relevant parameter which is frequently discussed in international literature as it represents an important pathologic articular morphology that is a relevant risk factor for patellofemoral instability[2, 3, 6, 11, 12, 14–17, 21].

One of the first studies using 3D imaging of the dysplastic trochlea was published by Biedert et al.[2]. In their study the authors showed that MRI 3D imaging was not only feasible, but they were able to identify variations in the dysplastic trochlea that were poorly represented using standard radiographs, CT scans, or routine 2D MRI imaging[2].

Fritz et al. postulated a higher proportion of correctly diagnosed cases of trochlear dysplasia after evaluation of 3D-printed models in comparison to CR/CT, and therefore, concluded that 3D models of the knee have the potential to improve diagnosis of patellofemoral dysplasia especially for less experienced surgeons [7]. The superiority of 3D representations has also been proven in other areas of medical practice. Wong et al. showed that 3D models of the hip joint can be beneficial for preoperative planning of femoroacetabular impingement surgery [29]. Another study demonstrated that 3D-printed models can precisely represent the size and shape of visceral aneurysms [23].

Apart from the analysis of the classification of trochlear dysplasia in 2D and 3D, another focus of this study was the distinction with regard to different classification options. In summary, the analysis carried out within this study shows a clear superiority of the 2-grade compared to the 4-grade-classification by Dejour.

Unsatisfactory results in the agreement of Dejour’s 4-grade classification were already reported in different publications [12, 21, 25, 28], why other classification-options presented in order to achieve more homogeneous results in the assessment of patellofemoral pathologies.

Biedert et al. [3] proposed significantly different trochlear medial and central condylar heights in patients with trochlear dysplasia [3].

Sharma et al. [21] developed a new classification system to assess the severity of trochlear dysplasia in axial MRI slices and demonstrated fair-to-good interobserver and good-to-excellent intraobserver agreement values, which, according to their classification, were found to be better than the Dejour classification on both CT and MRI [21].

Although interobserver and intraobserver agreements of other classification systems seem to be higher, Dejour’s classification can still be regarded as state of the art when evaluating trochlear dysplasia [9]. On the basis of the results obtained within this study as well as the studies of the current literature, however, the Dejour classification as basis for a therapy decision must be questioned. With the development, application and validation of software-generated 3D models, as used within this study, the development of new 3D-based classification systems for the assessment of patellofemoral pathologies should also be forced.

This study has several limitations. First, the number of raters was relatively small with no radiologists included. Second, the number of included MRI scans was relatively small and no MRI scans of patients without trochlear dysplasia were analyzed. Third, no learning effect was investigated as this was the first-time use of the developed 3D models for assessing the patellofemoral anatomy. Despite the above-mentioned limitations, in view of the complex anatomy and the data obtained, the 3D representation for assessing the patellofemoral anatomy can be regarded as beneficial with regard to the reliability in classification of trochlear dysplasia and so potentially eases an automated analysis of the present pathology as well as an individualized surgical planning that is aimed in the future. Further, a 3D-based classification system potentially would befit the superiority, demonstrated in this study, of 3D representation in the assessment of trochlear dysplasia.

## Conclusions

Overall, moderate-to-good agreement values were found in all groups. The analysis of intraobserver reliability showed no relevant differences between 2 and 3D representation, but better agreement values were found in the 2-degree classification compared to the 4-degree classification. With regard to the interobserver reliability, better agreement values were found in the 3D compared to the 2D representation. Therefore, in view data obtained within this study, the 3D representation for assessing the patellofemoral anatomy can be regarded as beneficial with regard to the reliability in classification of trochlear dysplasia, which is relevant for future analytical procedures as well as surgical planning.

## Data Availability

All relevant data are provided within the manuscript. The datasets used and/or analyzed during the current study are available from the corresponding author on reasonable request.
